# MAML1 Enhances the Transcriptional Activity of Runx2 and Plays a Role in Bone Development

**DOI:** 10.1371/journal.pgen.1003132

**Published:** 2013-01-10

**Authors:** Takashi Watanabe, Toshinao Oyama, Maki Asada, Daisuke Harada, Yoshiaki Ito, Masayo Inagawa, Yutaka Suzuki, Sumio Sugano, Ken-ichi Katsube, Gerard Karsenty, Toshihisa Komori, Motoo Kitagawa, Hiroshi Asahara

**Affiliations:** 1 Department of Systems BioMedicine, National Research Institute for Child Health and Development, Tokyo, Japan; 2 Department of Systems Biomedicine, Tokyo Medical and Dental University, Tokyo, Japan; 3 Department of Molecular and Tumor Pathology, Chiba University Graduate School of Medicine, Chiba, Japan; 4 Department of Medical Genome Sciences, Graduate School of Frontier Sciences, University of Tokyo, Kashiwa, Japan; 5 Department of Oral Pathology, Graduate School of Medical and Dental Sciences, Tokyo Medical and Dental University, Tokyo, Japan; 6 Department of Genetics and Development, Columbia University, New York, New York, United States of America; 7 Department of Cell Biology, Nagasaki University Graduate School of Biomedical Science, Nagasaki, Japan; 8 Department of Molecular and Experimental Medicine, The Scripps Research Institute, La Jolla, California, United States of America; Stanford University School of Medicine, United States of America

## Abstract

Mastermind-like 1 (MAML1) is a transcriptional co-activator in the Notch signaling pathway. Recently, however, several reports revealed novel and unique roles for MAML1 that are independent of the Notch signaling pathway. We found that MAML1 enhances the transcriptional activity of runt-related transcription factor 2 (Runx2), a transcription factor essential for osteoblastic differentiation and chondrocyte proliferation and maturation. MAML1 significantly enhanced the Runx2-mediated transcription of the p6OSE2-Luc reporter, in which luciferase expression was controlled by six copies of the osteoblast specific element 2 (OSE2) from the Runx2-regulated osteocalcin gene promoter. Interestingly, a deletion mutant of MAML1 lacking the N-terminal Notch-binding domain also enhanced Runx2-mediated transcription. Moreover, inhibition of Notch signaling did not affect the action of MAML1 on Runx2, suggesting that the activation of Runx2 by MAML1 may be caused in a Notch-independent manner. Overexpression of MAML1 transiently enhanced the Runx2-mediated expression of alkaline phosphatase, an early marker of osteoblast differentiation, in the murine pluripotent mesenchymal cell line C3H10T1/2. MAML1^−/−^ embryos at embryonic day 16.5 (E16.5) had shorter bone lengths than wild-type embryos. The area of primary spongiosa of the femoral diaphysis was narrowed. At E14.5, extended zone of collagen type II alpha 1 (Col2a1) and Sox9 expression, markers of chondrocyte differentiation, and decreased zone of collagen type X alpha 1 (Col10a1) expression, a marker of hypertrophic chondrocyte, were observed. These observations suggest that chondrocyte maturation was impaired in MAML1^−/−^ mice. MAML1 enhances the transcriptional activity of Runx2 and plays a role in bone development.

## Author Summary

To identify new molecules involved in bone and cartilage development and/or homeostasis, we utilized approximately 10,000 arrayed and addressable cDNA clones, which allowed systematic, efficient, and unbiased screening of cDNAs encoding factors that could activate critical bone differentiation activity via activation of Runx2, master regulator of bone development. We analyzed MAML1^−/−^ mice to investigate the role of MAML1 in bone development. MAML1^−/−^ embryos at embryonic day 14.5 and 16.5 had shorter bone lengths than wild-type embryos. The area of primary spongiosa of the femoral diaphysis was narrowed, indicated that chondrocyte maturation was impaired. This revealed that MAML1 plays an important role in proper bone development and may provide us with a new basis for identifying potential therapeutic targets for bone diseases.

## Introduction

Runt-related transcription factor 2 (Runx2) is a transcription factor belonging to the Runx gene family, which is homologous to *Drosophila runt*, a pair-rule gene involved in somitogenesis [1]. Runx2 is an essential factor for bone and hypertrophic cartilage formation that is expressed very early in bone development and continues to be present through the later phase of development [2]. Runx2 promotes the differentiation of pluripotent mesenchymal progenitor cells into the osteogenic lineage, but its role in terminal differentiation to mature osteoblasts and the production of bone matrix remains unclear. To date, it has been reported that several transcription factors and cofactors, such as TAZ [3], Grg5 [4], Rb [5], and HDAC4 [6], interact with Runx2 and positively or negatively regulate its function. However, in many cases, the physiological significance of the interaction is unclear. To further elucidate the function of Runx2, we performed luciferase assay-based screening of additional factors regulating the transcriptional activity of Runx2 using a full-length cDNA library containing approximately 10,000 clones. The screening system identified the mastermind-like (MAML) family of proteins showed especially strong potential for regulating Runx2 transcriptional activity. Overexpression of MAML1 enhanced the Runx2-mediated expression of alkaline phosphatase, an early marker of osteoblast differentiation, in C3H10T1/2 cells. Furthermore, MAML1^−/−^ embryos at E14.5 and 16.5 had shorter bone lengths than wild type embryos. The area of primary spongiosa of the femoral diaphysis was narrowed, indicated that chondrocyte maturation was impaired. These data suggest that MAML1 enhanced the transcriptional activity of Runx2 and plays a role in bone development.

## Results

### MAML1 enhances the transcriptional activity of Runx2

**Figure 1:**
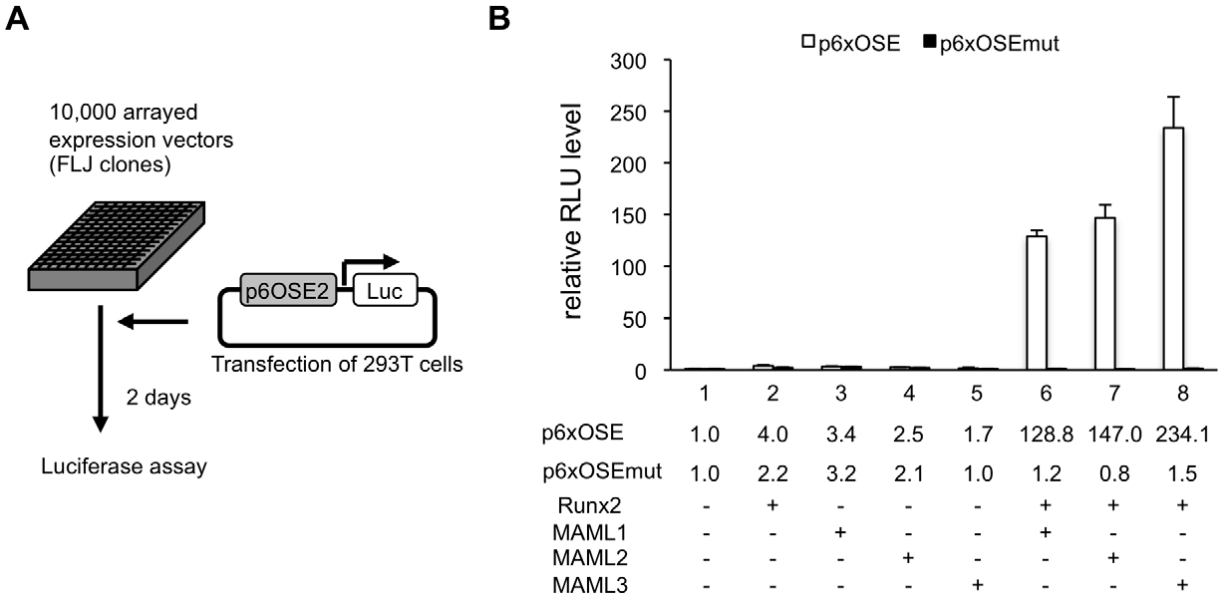
MAML1 enhances the transcriptional activity of Runx2. A, 293T cells were transiently transfected with p6OSE2-Luc reporter, Runx2 expression plasmid, and about 10,000 FLJ expression plasmids. B, 293T cells were transiently transfected with a p6OSE2-Luc reporter or p6OSE2-mutant-Luc together with MAML1, MAML2, MAML3, and Runx2 expression plasmids. Luciferase levels were normalized to the Renilla luciferase activity of a cotransfected phRL-TK-Luc reporter and presented as fold activation relative to the luciferase level of the p6OSE2-Luc reporter construct alone. Error bars represent the standard deviation of triplicate transfections.

We used a full-length cDNA library containing approximately 10,000 clones (FLJ clones, established by New Energy and Industrial Technology Development Organization [NEDO], Japan) and p6OSE2-Luc reporter assay system (Figure 1A). We identified a few novel factors that enhance Runx2 transcriptional activity. Among them, AK123604 (*Homo sapiens* cDNA FLJ41610), which is highly similar to Mastermind-like protein 3 (MAML3), showed especially strong activity. MAML is a human homolog of *Drosophila* mastermind, a protein that plays a role in the Notch signaling. MAML family members consist of MAML1, MAML2 and MAML3. We found that MAML1 and MAML2 also enhanced Runx2 transcriptional activity as well (Figure 1B). Because the establishment of knockout mice of MAML1 preceded MAML2 and MAML3, we primarily analyzed MAML1.

### MAML1 enhances Runx2 activity in a Notch-independent manner in vitro

**Figure 2:**
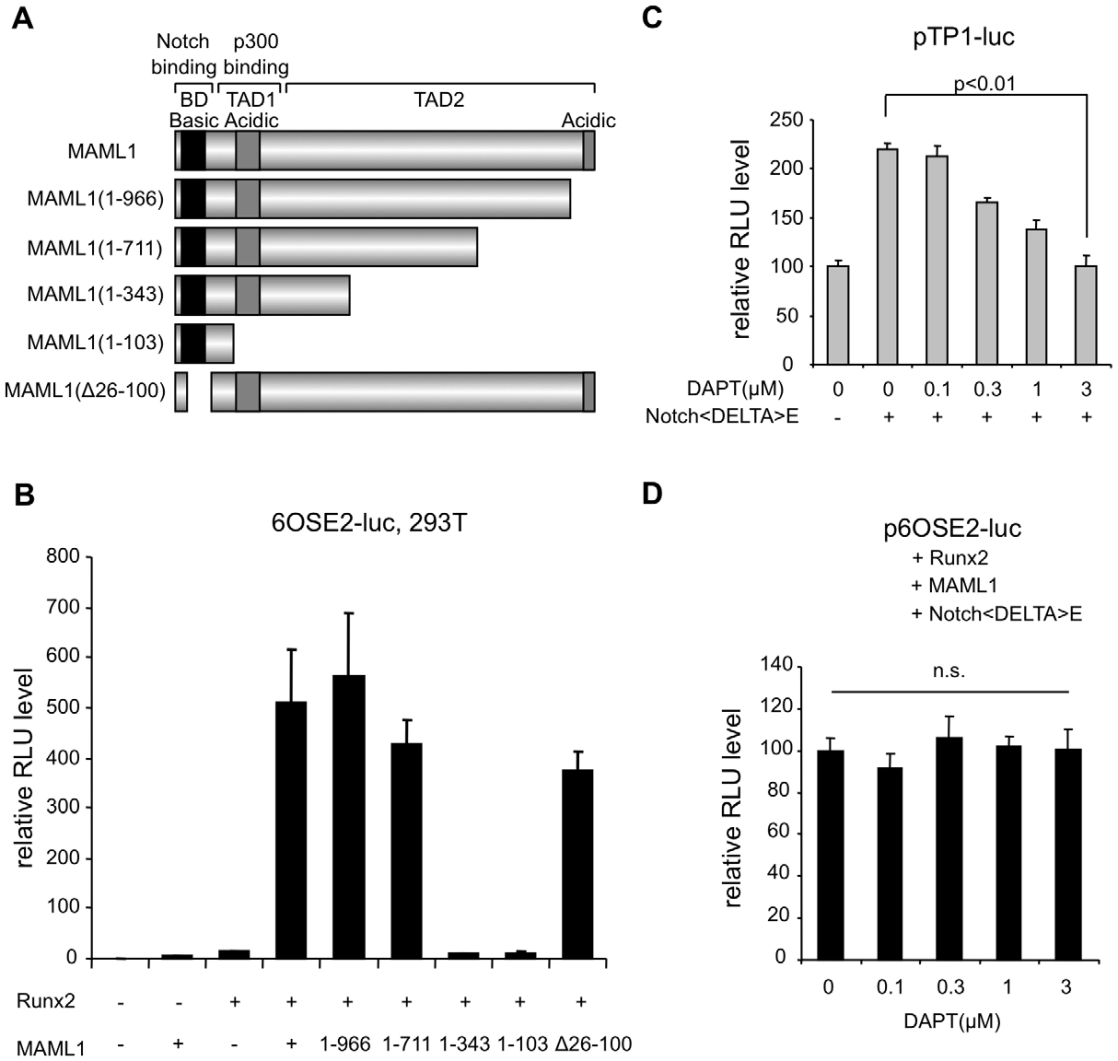
MAML1 enhances Runx2 activity in a Notch-independent manner in vitro. A, The structure of MAML1 and its truncated forms. B, 293T cells were transiently transfected with a p6OSE2-Luc reporter alone or together with truncated forms of MAML1 Error bars represent the standard deviation of triplicate transfections. C, 293T cells were transiently transfected with a pTP1-Luc reporter and NotchΔE expression plasmid in the presence of γ-secretase inhibitor (DAPT). Error bars represent the standard deviation of triplicate transfections. D, 293T cells were transiently transfected with a p6OSE2-Luc reporter with Runx2, MAML1 and NotchΔE expression plasmid in the presence of γ-secretase inhibitor (DAPT). Error bars represent the standard deviation of triplicate transfections.

MAML1 consists of 1016 amino acids and contains a conserved basic region and two acidic regions. To investigate which region is essential for regulating Runx2, we assessed each deletion mutant by p6OSE2-Luc reporter assay (Figure 2). The N-terminal basic region, which is essential for the interaction with Notch, and the C-terminal acidic region of MAML1 were dispensable for Runx2 transcriptional activity. On the other hand, the center region (residues 343–711), whose function is not well known, was essential.

Because MAML1 is a coactivator of Notch signaling, we investigated whether or not the action of MAML1 on Runx2 was dependent on Notch. Notch1ΔE is cleaved by γ-secretase to produce the Notch intracellular domain (NICD), which translocates into the nucleus and transactivates the target gene. A *Γ*-secretase inhibitor DAPT inhibited the Notch1ΔE-mediated activation of pTP1-Luc, in which luciferase expression was controlled by Notch signaling (Figure 2C). On the other hand, DAPT did not affect the action of MAML1 on Runx2 in the presence of Notch1ΔE (Figure 2D). This suggests that MAML1 possibly enhance the transcriptional activity of Runx2 in a Notch-independent manner.

### MAML1 promotes Runx2-mediated osteoblastic differentiation

**Figure 3:**
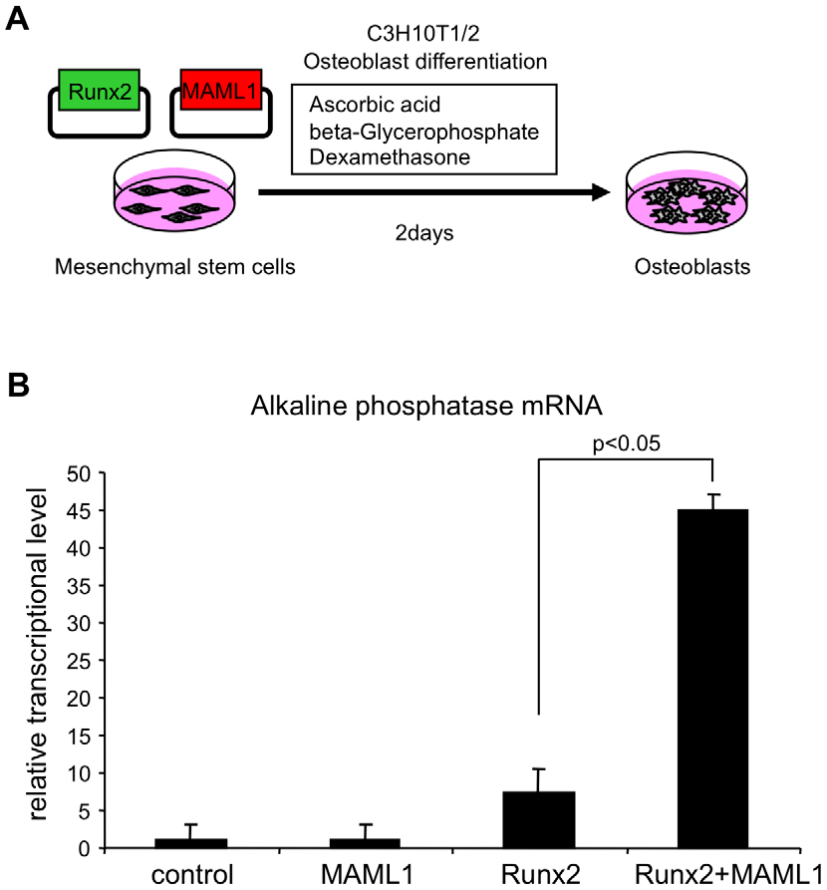
MAML1 promotes Runx2-mediated osteoblastic differentiation. A, C3H10T1/2 cells were transiently transfected with a Runx2 and/or MAML1 expression plasmids and cultured for 2 days. B, Total RNA was isolated and reverse-transcribed and TaqMan real-time PCR was performed to investigate the expression level of alkaline phosphatase gene, a marker of osteoblast.

293T cell used in the luciferase assay is derived from human embryonic kidney and does not express Runx2. Therefore, we next investigated whether MAML1 controls osteoblastic differentiation through Runx2 in the murine pluripotent mesenchymal cell line C3H10T1/2 (Figure 3). Overexpression of Runx2 promoted the expression of the ALP gene, an early osteoblast marker. Co-overexpression of MAML1 rapidly augmented the Runx2-mediated expression of ALP, whereas MAML1 alone did not induce ALP expression. However, this effect was observed only in early phase of osteoblast differentiation and later phase markers such as bone sialoprotein and osteocalcin were not changed (data not shown).

### Analysis of skeletal defects in MAML1^−/−^ mice

**Figure 4:**
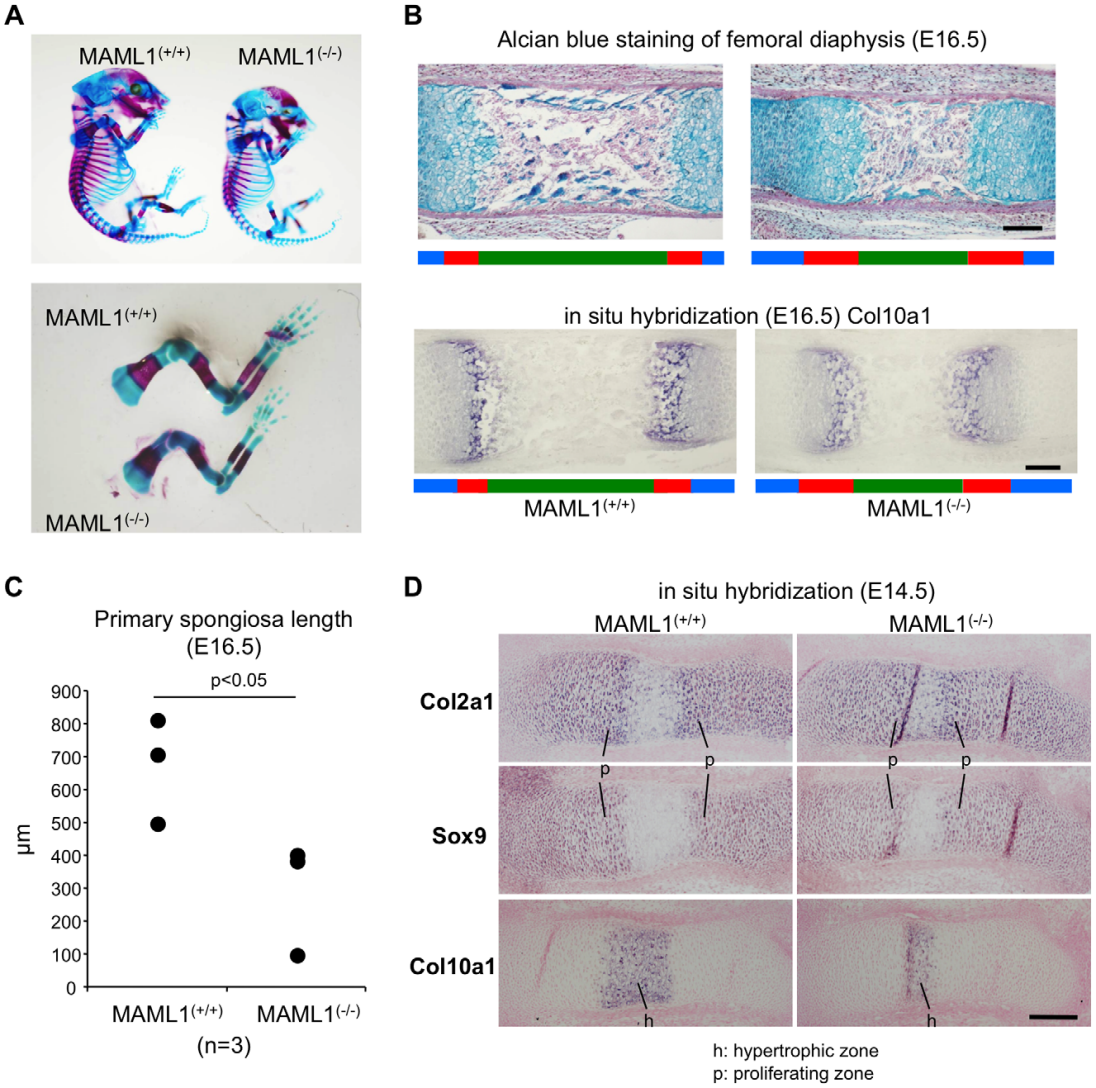
Analysis of skeletal defects in MAML1^−/−^ mice. A, Whole mounted embryos at E16.5 were stained with Alcian Blue and Alizarin Red. B, The femoral sections at E16.5 were stained with Alcian Blue (upper panel). Arrow bar indicates the area of primary spongiosa. The expression of collagen, type 10 alpha 1, a marker of hypertrophic chondrocyte, was shown by in situ hybridization (lower panel). Green bars, primary spongiosa; red bars, hypertrophic zone; blue bars, proliferating zone. Black bars, 200 µm. C, The primary spongiosa length of MAML1 null (KO) embryos and wild type (WT) littermates. D. Femoral sections at E14.5. The expression of type 10 alpha 1 collagen, type 2 alpha 1 collagen and sox9 was shown by in situ hybridization. Bars, 100 µm.

We analyzed MAML1 knockout (MAML1^−/−^) mice [7]. Normal Mendelian ratios are observed up to E18.5, but MAML1^−/−^ mice with C57BL/6 background die during the perinatal period. In the original paper, however, MAML1−/− mice die within 14 days after birth [7]. The difference of lethality in the mice is thought to be due to the difference of the background. At E16.5, MAML1^−/−^ mice were smaller than wild type mice (Figure 4A). Whole mounted embryos at E16.5 stained with Alcian Blue and Alizarin Red showed that the mineralized region in the long bones of MAML1^−/−^ mice was relatively short compared with wild type mice (Figure 4A). Histological analysis revealed that the area of primary spongiosa of the femoral diaphysis was reduced in MAML1^−/−^ mice compared to wild type mice (Figure 4B, 4C). At E14.5, extended zone of Col2a1 and Sox9 expression, markers of chondrocyte differentiation, and decreased zone of Col10a1 expression, a marker of hypertrophic chondrocyte, were observed (Figure 4D). These observations indicated the impairment of chondrocyte maturation in MAML1^−/−^ mice.

## Discussion

We utilized approximately 10,000 arrayed and addressable cDNA clones, which allowed systematic, efficient, and unbiased screening of cDNAs encoding factors that could activate Runx2-mediated expression of the p6OSE2-Luc reporter construct (Figure 1). This revealed that MAML was a potential activator of Runx2-mediated luciferase expression.

MAML is a coactivator of Notch signaling. Upon ligand stimulation from neighboring cells, Notch is cleaved by γ-secretase and its intracellular domain (NICD) translocates into the nucleus [8]. NICD interacts with CSL through a RAM domain at the N-terminus that has high affinity for the β-trefoil domain of CSL. Then, the ankyrin repeats domain of NICD docks with the Rel-homology domain of CSL and creates a high-affinity binding site for MAML. MAML associates with the CSL-NICD complex through the N-terminal basic region, recruits p300, RNA polymerase II and other unknown factors, and activates the transcription of target genes such as Hes1 [9], [10]. On the other hand, Notch-independent action of MAML1 on p53 [11], beta-catenin [12], MEF2C [13] and NF-kappaB [14] has been previously reported.

Recently, two groups have published studies using genetically modified mice [15], [16]. Hilton and colleagues showed that Notch signaling inhibits osteoblast differentiation through Hes or Hey proteins, which diminish Runx2 transcriptional activity via physical interaction, and acts to maintain a pool of mesenchymal progenitors. Engin and colleagues showed that pathological gain of Notch function in established osteoblastic lineages activates expansion of the immature osteoblastic pool by increasing transcription of the genes encoding osterix, cyclin D and cyclin E and by repressing the function of Runx2 by direct interaction and inhibition of its binding. These findings suggest that Notch signaling negatively regulate the function of Runx2. We indicated that the N-terminal Notch-binding region of MAML1 is dispensable for the action of MAML1 on Runx2 and Notch signaling inhibitor does not affected the action of MAML1 on Runx2. Furthermore, knockdown of p300, a coactivator [9], [10], did not affected the activation of Runx2 transcriptional activity by MAML1 (data not shown). These data suppose that the action of MAML1 on Runx2 is Notch-independent.

To elucidate how MAML regulates Runx2-mediated transcription, we investigated the physical interaction of MAML1 with Runx2, but we could not demonstrate the interaction between Runx2 and MAML1 (data not shown), suggesting that this interaction is very weak and possibly indirect.

We showed the impairment of chondrocyte maturation in MAML1^−/−^ mice. Because Runx2 facilitates chondrocyte maturation, the phenotype of MAML1^−/−^ mice may be caused by the dysfunction of Runx2. On the other hand, the expression of Sox9, a transcription activator of collagen type II, was upregulated by Notch activation and this activation of Notch signaling thereby promoted differentiation of proliferative and prehypertrophic chondrocytes [17]. Therefore, from this current findings, it is not clear yet whether or not the phenotype of MAML1^−/−^ mice is due to the dysfunction of Runx2 or Notch signaling. Other possibility to explain MAML1^−/−^ mice bone phenotype is that other cell signaling cascades and molecules could be involved into MAML dependent gene regulation and thus bone development. For example, MEF2C, a transcription factor that regulates muscle and cardiovascular development, was reported to control bone development by activating the gene program for chondrocyte hypertrophy [18].

Taken together, our analysis revealed novel function of MAML1, Notch independent promotion of Runx2 activity and its role in bone development. Further elucidation of the precise molecular mechanisms responsible for the initiation and termination of this functional association during bone development may provide us with a new basis for understanding the molecular network in osteoblasts and potential therapeutic targets for bone diseases.

## Materials and Methods

### Ethics statement

All animal experiments were performed according to protocols approved by the Institutional Animal Care and Use Committee at National Institute for Child Health and Development (Protocol 2004-003).

### Plasmids

The p6OSE2-Luc and p6OSE2-mut-Luc reporter construct were previously reported [19]. The pEF-BOS hMam-1 (MAML1) plasmid, its truncated forms [20], hMam-2 (MAML3), hMam-3 (MAML2) [21] and pCS2+Notch1ΔE [22] were previously reported. The pCG mRunx2 plasmid by Dr. Nakashima (Tokyo Medical and Dental University, Tokyo), and the p3xFLAG mRunx2 plasmid by Dr. Hikata (Keio University, Tokyo). The pTP1-Luc (pGa981-6) construct was provided by Dr. Ursula Strobl (Institute of Clinical Molecular Biology and Tumor Genetics, Germany).

### Reporter assays

For the primary screening, we diluted approximately 10,000 FLJ clones (Full-length human cDNA sequencing project, NEDO) to 10 ng/µL in 10 mM Tris-HCl (pH 8.5), and dispensed 5 µL to each well in 384-well plates. We then added 10 ng of p6OSE2-Luc, 2 ng of pCG, 0.1 µL of Fugene6 (Roche Diagnostics), and 5 µL of Opti-MEM I Reduced-Serum Medium (Invitrogen) to each well. 293T cells were diluted to 1.25×10^5^ cells/mL with Dulbecco's modified Eagle medium (DMEM) containing 10% heat-inactivated fetal bovine serum (FBS), 50 units/mL penicillin, and 50 µg/mL streptomycin, and seeded at 40 µL (5,000 cells) per well. After 48 hours of culture, we removed the supernatant and added 40 µL of Steady Glo Luciferase Assay Reagent (Promega) diluted 2-fold with phosphate buffered saline (PBS) to each well. After 10 minutes at room temperature, luminescence was measured using a plate reader (ARVO, Perkin Elmer). After the second screening, the assay was performed in 96-well plates. We added γ-secretase inhibitor IX, N-[N-(3, 5-difluorophenylacetyl-L-alanyl)]-S-phenylglycine t-butylester (DAPT; Calbiochem), to the medium 2 hours before transfection.

### Cell culture, transfection, and differentiation assays

We purchased the C3H10T1/2 murine pluripotent mesenchymal cell line from ATCC and maintained it in DMEM containing 10% heat-inactivated FBS, 50 units/mL penicillin, and 50 µg/mL streptomycin. For differentiation assays, we seeded cells in a multi-well plate at a density of 2,000 cells/cm^2^ and cultured them for 3 days. The medium was then changed to the osteoblastic medium (MEM-alpha containing heat-inactivated FBS, 50 units/mL penicillin, 50 µg/mL streptomycin, 50 µM ascorbic acid 2-phosphate, 10 mM β-glycerophosphate, and 0.1 µM dexamethasone), transfected with p3xFLAG-Runx2 and/or pEF BOS-hMam1 by FugeneHD (Roche diagnostics), and cultured.

### Real-time PCR

We isolated total RNA from the cultured cells using the RNeasy mini kit (QIAGEN) and reverse transcribed 2 µg of total RNA using Ready-To-Go You-Prime First-Strand Beads (GE Healthcare) and oligo-dT primer. The products were diluted 10-fold with distilled water and used as a template for real-time PCR. Real-time PCR was performed using a TaqMan Gene Expression Assay, TaqMan Universal PCR Mix and the 7900HT Fast Real-Time PCR System (Applied Biosystems).

### Double staining of MAML1^−/−^ mouse embryos

We backcrossed MAML1 null mice [7] at least 10 times onto a C57BL/6 background. We fixed mouse embryos on embryonic day 16.5 (E16.5) in ethanol overnight and then stained them overnight with Alcian blue solution (0.15 mg/mL Alcian blue 8GX in 20% acetic acid and 80% ethanol). The embryos were washed briefly with ethanol twice, treated with 2% potassium hydroxide overnight, and then stained overnight with Alizarin red solution (0.075 mg/mL Alizarin red S in 1% potassium hydroxide).

### Alcian blue staining

Tissues were fixed in 4% paraformaldehyde-PBS overnight at 4°C, processed, embedded in paraffin, and sectioned. Slides were deparaffinized, washed with water, treated with 3% acetic acid, and then with 1% Alcian blue 8GX for 60 minutes. After staining, we washed the slides briefly with 3% acetic acid, then with water for 5 minutes, counterstained with Kernechtrot Stain Solution (Muto Pure Chemicals, Tokyo) for 5 minutes, washed with water for 3 minutes, and dehydrated the slides.

### In situ hybridization

Tissues were fixed in 4% paraformaldehyde-PBS overnight at 4°C, processed, embedded in paraffin, and sectioned. Slides were deparaffinized, treated with proteinase K (8 µg/mL) for 10 minutes at RT, and then with 0.2% glycine in PBS for 10 minutes at RT. Slides were refixed in 4% paraformaldehyde-PBS for 10 minutes at RT, washed with PBS for 5 minutes 3 times, acetylated with 0.1 M triethanolamin-HCl (pH 8.0) for 10 minutes, washed with PBS for 30 minutes, and then prehybridized with prehybridization buffer (50% deionized formamide and 5× saline-sodium citrate (SSC)) for 60 minutes at 65°C. We hybridized the slides with DIG-labeled antisense riboprobes in hybridization buffer (50% deionized formamide, 5× SSC, 0.25 mg/mL yeast tRNA, 10% dextran sulfate, and 5× Denhardt's solution) in a humidified chamber at 65°C overnight. After hybridization, the slides were washed with 5× SSC (1× SSC: 0.15 M NaCl, 0.015 M sodium citrate) at 65°C for 20 minutes, 0.2× SSC at 65°C for 3 hours, and NT buffer (0.1 M Tris-HCl [pH 7.5], 0.15 M NaCl) for 5 minutes at RT. We incubated the slides at 4°C overnight with alkaline phosphatase (ALP)-coupled anti-DIG antibody in NT buffer containing 0.1% sheep serum. The slides were washed with NT buffer for 15 minutes 3 times and equilibrated in NTM (0.1 M NaCl, 0.1 M Tris-HCl [pH 9.5], and 0.05 M MgCl_2_) for 5 minutes at RT. The slides were then treated with BM Purple AP Substrate (Roche) for 3 hours at RT in a humid chamber protected from light.

### Statistical analysis

The two-tailed independent Student's *t-*test was used to calculate all *P* values.
